# Acute adaptive immune response correlates with late radiation-induced pulmonary fibrosis in mice

**DOI:** 10.1186/s13014-015-0359-y

**Published:** 2015-02-20

**Authors:** Alexandra Paun, Amit Kunwar, Christina K Haston

**Affiliations:** Department of Human Genetics, Meakins-Christie Laboratories, McGill University, Montreal, QC Canada; Department of Medicine, Meakins-Christie Laboratories, McGill University, 3626 St. Urbain, H2X 2P2 Montreal, QC Canada

**Keywords:** Fibrosis, Strain-dependent radiation response, Inbred strains, Cytokines, Late effects

## Abstract

**Background:**

The lung response to radiation exposure can involve an immediate or early reaction to the radiation challenge, including cell death and an initial immune reaction, and can be followed by a tissue injury response, of pneumonitis or fibrosis, to this acute reaction. Herein, we aimed to determine whether markers of the initial immune response, measured within days of radiation exposure, are correlated with the lung tissue injury responses occurring weeks later.

**Methods:**

Inbred strains of mice known to be susceptible (KK/HIJ, C57BL/6J, 129S1/SvImJ) or resistant (C3H/HeJ, A/J, AKR/J) to radiation-induced pulmonary fibrosis and to vary in time to onset of respiratory distress post thoracic irradiation (from 10–23 weeks) were studied. Mice were untreated (controls) or received 18 Gy whole thorax irradiation and were euthanized at 6 h, 1d or 7 d after radiation treatment. Pulmonary CD4+ lymphocytes, bronchoalveolar cell profile & cytokine level, and serum cytokine levels were assayed.

**Results:**

Thoracic irradiation and inbred strain background significantly affected the numbers of CD4+ cells in the lungs and the bronchoalveolar lavage cell differential of exposed mice. At the 7 day timepoint greater numbers of pulmonary Th1 and Th17 lymphocytes and reduced lavage interleukin17 and interferonγ levels were significant predictors of late stage fibrosis. Lavage levels of interleukin-10, measured at the 7 day timepoint, were inversely correlated with fibrosis score (R = −0.80, p = 0.05), while serum levels of interleukin-17 in control mice significantly correlated with post irradiation survival time (R = 0.81, p = 0.04). Lavage macrophage, lymphocyte or neutrophil counts were not significantly correlated with either of fibrosis score or time to respiratory distress in the six mouse strains.

**Conclusion:**

Specific cytokine and lymphocyte levels, but not strain dependent lavage cell profiles, were predictive of later radiation-induced lung injury in this panel of inbred strains.

**Electronic supplementary material:**

The online version of this article (doi:10.1186/s13014-015-0359-y) contains supplementary material, which is available to authorized users.

## Introduction

The radiation-induced lung injury is thought to occur through the ionizing radiation producing reactive oxygen species which induce lesions in DNA leading to damage of the alveolar epithelium [1] and capillary endothelium [2]. The inflammatory reaction to cell damage, including lymphocyte [3] and neutrophil [4] recruitment, if excessive or sustained, likely through epithelial [5] and leukocyte cell [3] derived cytokine signalling, can lead to the overwhelming inflammatory response of alveolitis/pneumonitis. The tissue response to injury can also be resolved as fibrosis, which is characterized by the progressive accumulation of extracellular matrix constituents replacing normal functional parenchyma [6] and may feature bone marrow cell recruitment [7,8].

Assays of radiation-induced clinical injury, such that susceptible persons environmentally or therapeutically exposed can be predicted before the onset of symptoms, have been sought. Initial radiation response assays such as chromosomal aberrations, DNA damage and cell survival in cultured fibroblasts or lymphocytes offered conflicting results between *in vitro* cellular radiosensitivity and *in vivo* normal tissue response [9-12] suggesting that intrinsic radiosensitivity at the cellular level is not the only determinant for radiotherapy side-effects. Part of the tissue injury response to the primary radiation damage is a continuum of cytokine-based, multicellular interactions [13]. This has prompted the study of early circulatory markers as predictive assays and has revealed associations of serum interleukins (Il6, Il1a, Il8, Il10) with pneumonitis [14-17]. None of these markers, however, robustly correlates with late tissue injury supporting the notion that the late stage response is dictated by a complex cytokine cascade which develops following the initial injury [18,19].

The differential radiation response of inbred strains of mice has been used as a model of radiation induced lung injury [20-22]. For example, our studies indicated that following a dose of 18 Gy to the lung, mice of a panel of evaluated inbred strains (n = 27 strains) developed significant pneumonitis, but differed in the time post irradiation at which respiratory distress from this condition developed [22]. Mice of the KK/HIJ, C3H/HeJ and AKR/J strains presented distress at 10–14 weeks after radiation treatment while this trait was evident in C57BL/6J, 129S1/SvImJ and A/J mice at 21–23 weeks after 18 Gy. The inbred strains also differed in extent of pulmonary fibrosis evident at distress [22,23], or their fibrosis score. Specifically, KK/HIJ, C57BL/6J, 129S1/SvImJ mice all had significant pulmonary fibrosis, with pneumonitis, when in distress and mice of the C3H/HeJ, A/J and AKR/J strains succumbed with pneumonitis and no fibrosis.

In addition to histological features of disease, mice respond to thoracic radiation with a lymphocytic infiltrate [24-26] which is consistent with clinical findings [3,27]. Perhaps more relevant than the presence of lymphocytes in the irradiated lung is their profile and repertoire of secreted mediators. Indeed, T helper (Th) cells in particular drive the inflammatory and fibrotic responses following injury through their production of cytokines. Specifically, the pro-inflammatory Th1 cells (CD4 + Ifnγ+) oppose the pro-fibrotic Th2 lymphocytes (CD4 + IL13+) [28], whereas Th17 cells (CD4 + IL17+) have emerged as potent inducers of inflammation and auto-immunity [29]. The Thelper response to thoracic irradiation of different lines of mice has not been reported, but mouse strains have been shown to differ in their cytokine response post radiation [24,30] and in gene expression profiles suggestive of differing adaptive immune responses [31]. Finally, significant heterogeneity in the pulmonary lymphocyte populations of inbred stains of mice has been reported [32]. These differences permit the identification of potentially novel biomarkers, which vary in induction by radiation exposure and are correlated to extent of lung disease, to be investigated in animal models.

Herein we aimed to determine whether the initial radiation response, assayed by pulmonary T helper and lavage cell counts and cytokine levels, is predictive of the lung injury responses of time to develop respiratory distress (pneumonitis) or of fibrosis in irradiated mice.

## Methods

### Mice

Female mice of six inbred strains (AKR/J, C3H/HeJ, A/J, C57BL/6J, 129S1/SvImJ, KK/HlJ) were purchased from the Jackson Laboratory (Bar Harbor, USA) and housed in the animal facility of the Meakins-Christie Laboratories. All mice were handled according to guidelines and regulations of the Canadian Council on Animal Care. Late stage radiation-induced phenotype values of pulmonary fibrosis score and time to respiratory distress, for these six strains, were taken from a previous report [22]. In that work mice received 18 Gy whole thorax irradiation and were euthanized upon presentation of respiratory distress which was defined as a loss of body weight exceeding 20%, accompanied by hunched posture, ruffled fur and visibly accelerated breathing. All mice euthanized due to presentation of distress symptoms had developed significant pneumonitis upon histological evaluation of lung tissue. The percent of the histological section covered with a fibrotic lesion was computed with imaging analysis as the fibrosis score of a mouse. The fibrosis scores used here are the average scores of mice from the same strain.

### Radiation exposure

Eight week old female mice were anesthetised with an intraperitoneal injection of sodium pentobarbital (30 mg/kg), partially shielded with 3 cm of lead and received whole thorax radiation exposure (18 Gy; dose rate 0.54 Gy/minute) using a Faxitron X ray machine. After irradiation, the animals were housed under normal laboratory conditions, and groups of 10 mice per strain were euthanized at 6 h, 1d or 7d after irradiation. 10 untreated mice of each strain served as controls. The control mice of each strain were not anaesthetized or irradiated and were euthanized at the 7 day time point. The mice were divided into 2 groups: one group was assayed by flow cytometry and the second was designated for bronchoalveolar lavage and serum collection.

### Serum collection

At necropsy, cardiac puncture was performed and >500 μl of blood was obtained. The blood was allowed to clot at room temperature for 1 h and centrifuged at 1500 g for 10 minutes. The serum was collected and stored at −80 degrees C until analysis.

### Bronchoalveolar lavage fluid collection

At the time of sacrifice BAL was collected with an injection of 1 ml PBS. The BAL volume recovered from each animal was recorded and the number of cells in this volume was determined using a hemocytometer. After centrifugation of the sample, the supernatant was stored and the pellet was resuspended in 100 μl of PBS and used for cell counting after staining with hematoxylin-eosin (Hema 3 Staining System, Fisher Diagnostics). Numbers of lymphocytes, macrophages and polymorphonuclear cells were morphologically identified from 500 counted cells per lavage sample and the total number of each cell type calculated from the percentage within the 500 cells and the number of cells/ml recovered from each animal.

### Cytokine measurement

Cytokines (Il6, Il4, Il1β, Il10, Il13, Il17, Ifnγ and Tnfα) were evaluated with a Bio-Rad mouse cytokine 8-plex panel run according to the manufacturer's instructions.

### Lymphocyte profiling

Total lung tissue was digested (37 degree C, 45 minutes) with 5 mg/ml DNase/collagenase (Roche Diagnostics) and a single cell suspension was obtained by mechanical dissociation of the tissue. The cells were counted using a trypan blue exclusion assay and 10^6^ total lung cells from each mouse were used for the flow cytometry assay. Total lung cells were stained with the surface antibody CD4 APC (eBioscience). Prior to intracellular staining the cells were stimulated with 50 ng/ml PMA (Sigma-Aldrich), 1 μg/ml Ionomycin (Sigma-Aldrich) and 1 μl/ml Golgi Stop (BD Biosciences). After permeabilization with Cytofix/Cytoperm (BD Biosciences), staining with intracellular antibodies (eBioscience) against Ifnγ, Il13 and Il17 was performed. Cell acquisition was completed using LSRII cytometer.

### Statistical analysis

The results are presented as mean ± SE of cell counts or cytokine measures of each strain at each time point. The statistical significance of the variability among the means, i.e. strain dependence of the phenotype, was determined by one way ANOVA. Correction for multiple testing was performed using the Bonferonni method by dividing the α = 0.05 significance threshold by the number of tests performed (i.e. 3 in the case of lung CD4+ lymphocyte analysis and bronchoalveolar lavage cell differentials, and 8 in the analysis of serum and lavage cytokines). The correlation tests were performed using the cor.test function in R (http://cran.r-project.org). Multiple linear regression was performed using the lm function in R and the phenotypes of percent fibrosis and of post irradiation survival time were evaluated as a function of each of BAL cell differentials, specific BAL and serum cytokines, and CD4 cell counts. These analyses were completed for each of the 4 time points, using the data from individual mice, i.e. not the average values for the 6 strains. Through multiple linear regression analyses we evaluated whether statistical models which included defined sets of cytokine or cell count variables, and terms permitting an interaction between variables to be assessed, were predictive of the lung responses of pulmonary fibrosis or respiratory distress. In this analysis the phenotype values (fibrosis score or post irradiation survival time) were evaluated as a function of the predictor variables (cytokines or cell counts) and their interaction using the following equation: phenotype = β0 + β1*variable1 + β2* variable2 + β3*interaction. The maximum likelihood estimators, βi, were calculated and given the null hypothesis of βi = 0, the predictor variable i (or the interaction term) was identified as a significant predictor of the phenotype values where βi was significantly different from 0.

## Results

### Acute Pulmonary CD4+ lymphocyte response to thoracic irradiation

**Figure 1:**
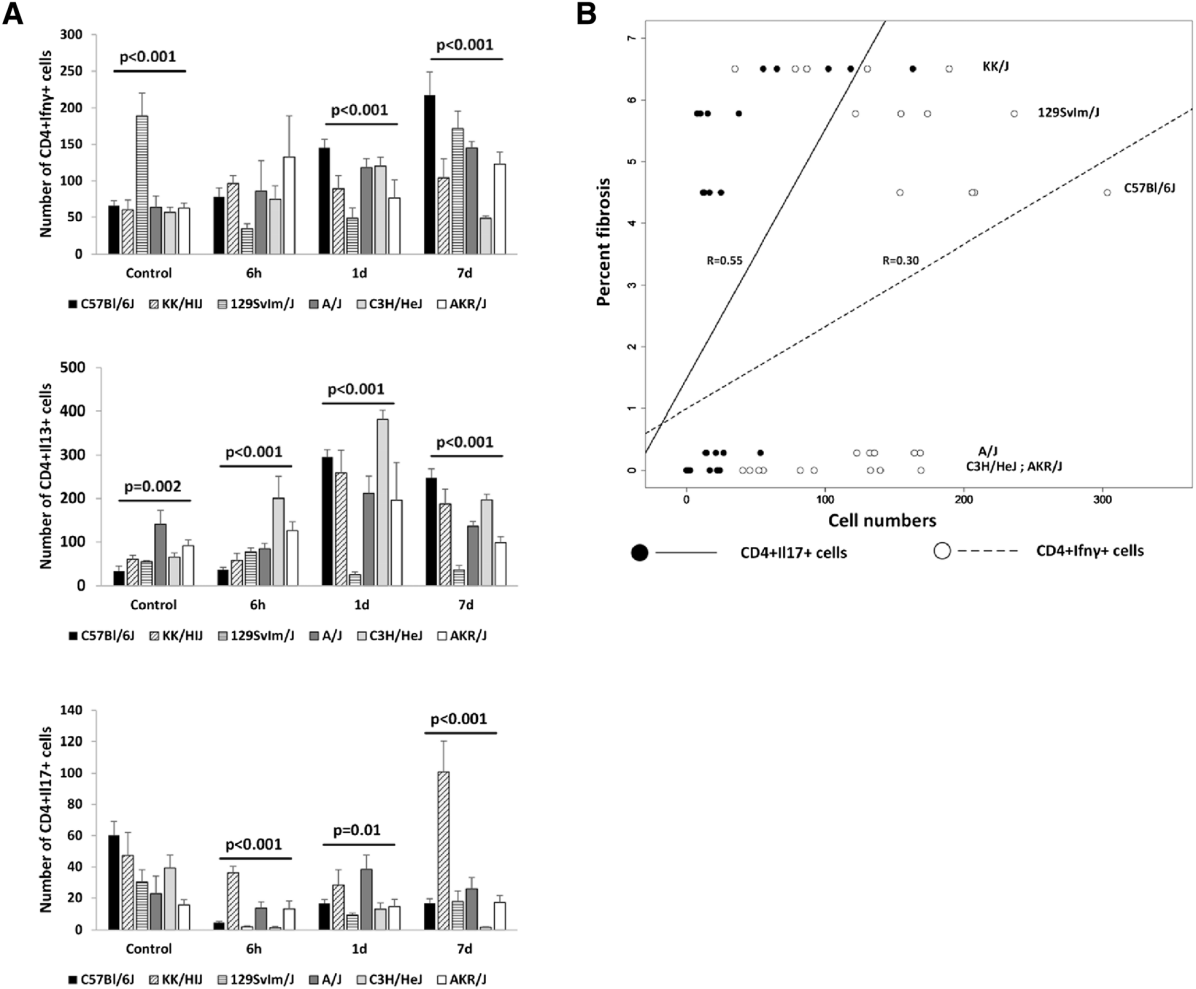
**Strain dependent pulmonary T helper cell populations in acute radiation response.** Mice were exposed to 18 Gy of thorax irradiation and euthanized at 6 hours, 1 day and 7 days post treatment. **A)** Th1 (CD4 + Ifnγ+), Th2 (CD4 + Il13+) and Th17 (CD4 + Il17+) cell counts were measured by flow cytometry. Mean ± SE of T helper cells among 10^6^ total lung cells, for groups of 4–5 mice. Significant variation in inbred strain measures by ANOVA indicated. **B)** Linear regression of the pulmonary fibrosis score against the numbers of CD4 + Ifnγ + and CD4 + Il17+ cells recorded at the day 7 time point. A significant interaction (p = 0.04) between the CD4 + Il17+ and CD4 + Ifnγ + cell counts is indicated by the intersecting regression lines of the fibrosis score against each individual variable.

Thoracic irradiation can induce a tissue injury response which includes increases in lymphocytes. To investigate whether numbers of distinct Thelper lymphocyte populations, assayed during the acute response to irradiation, could predict for the onset of respiratory distress or for the development of radiation-induced pulmonary fibrosis, we measured the CD4+ lymphocyte numbers in the lungs of strains of mice we had previously determined to vary significantly in the development of these traits [22,23,25,31,33-36]. Mice were irradiated with 18 Gy to the thorax, euthanized at 6 hours, 1 day or 7 days post irradiation and lymphocyte populations of the lung assessed with fluorescence activated cell sorting (Figure 1; gating strategy is provided in Additional file 1: Figure S1). The effect of radiation exposure on the numbers of CD4 + Il13+, CD4 + Ifnγ + and CD4 + Il17+ cells in the lungs was varied, as illustrated in Additional file 2: Figure S2, and no pattern of acute response common to fibrosis prone mice or fibrosis resistant mice, was evident. Inbred strain background, however significantly affected the numbers of CD4 + Il13+, CD4 + Ifnγ + and CD4 + Il17+ cells at each of the timepoints evaluated (p ≤ 0.01), as illustrated in Figure 1A, with the exceptions of CD4 + Il17+ cells at control (p = 0.07) and CD4 + Ifnγ + cells at 6 h (p = 0.33).

To determine whether the acute response CD4+ cell profile predicted for late stage lung disease, we analyzed the correlation of T helper cell numbers with the fibrosis score and post-irradiation survival time (hereafter survival time) previously documented for these strains of mice. Suggestive correlations between the numbers of CD4 + Il13+ cells at 6 h with fibrosis (R = −0.71, p = 0.1, an inverse correlation), and the numbers of CD4 + Ifnγ + cells at 7 days with survival time (R = 0.75, p = 0.08), were revealed, as shown in Additional file 3: Figure S3. The remaining potential correlations of lymphocyte number to fibrosis or survival time were not significant (p > 0.21).

Secondly, to investigate whether the pulmonary profile of CD4 + Il13+, CD4 + Ifnγ + and CD4 + Il17+ cell numbers at any of the assay times was, collectively, predictive of later lung disease we performed multiple linear regression analyses on these data. This analysis revealed the numbers of CD4 + Ifnγ + cells (p = 0.02), CD4 + Il17+ cells (p = 0.013), at the 7 day time point, and a model including an interaction between these two variables (p = 0.04) to significantly correlate with fibrosis score (R = 0.67; p = 0.003), as illustrated in Figure 1B. The model including an interaction of CD4 + Ifnγ + and CD4 + Il17+ cells was revealed to better explain the fibrosis score than models of CD4 + Il17+ or CD4 + Ifnγ + cell numbers alone: (p = 0.04 and p = 0.001 respectively, by ANOVA). The addition of CD4 + Il13+ cell counts to this model did not improve the correlation to fibrosis score (p = 0.27) and cell counts at the remaining time points were not significantly correlated to the fibrosis phenotype, (p > 0.18). Finally, no significant correlations of collective pulmonary CD4+ cell counts to survival time were revealed by multiple linear regression analyses (p > 0.32).

### Acute Bronchoalveolar lavage cellular response to thoracic irradiation

To investigate whether radiation exposure induced a strain dependent bronchoalveolar lavage cell count or profile, mice of 6 inbred strains were irradiated and lavage tissue procured at each of three times post treatment. Radiation exposure led to a difference among strains in total cell counts at 6 h and 7 days post-irradiation (ANOVA p < 0.002), as shown in Additional file 4: Figure S4A. To measure the association with late stage disease, we performed correlation tests which revealed a suggestively significant correlation of the total cell counts at 6 h post irradiation with fibrosis score (R = 0.77, p = 0.07, Additional file 4: Figure S4B), while the counts at 7 days were not correlated with either of fibrosis or survival time (p > 0.22, data not shown).

**Figure 2:**
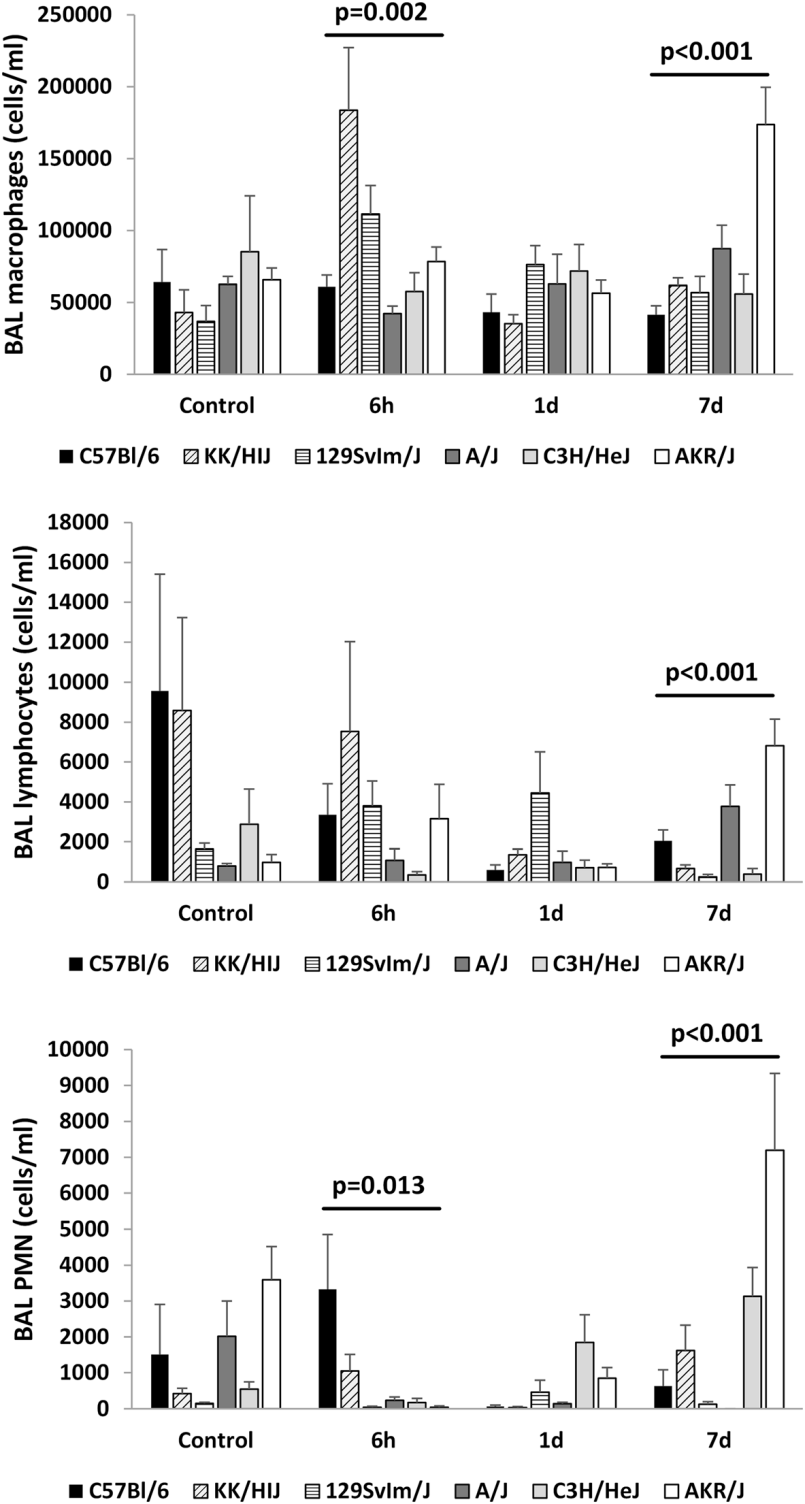
**Strain dependent bronchoalveolar lavage cell differentials in acute radiation response.** Bronchoalveolar lavage fluid was evaluated in control and irradiated mice 6 hours, 1 day and 7 days post-irradiation. Shown are the numbers of each cell type per ml of bronchoalveolar lavage fluid. Results are presented as mean ± SE for groups of n = 4–5 mice. Significant variation in inbred strain measures by ANOVA indicated.

In terms of bronchoalveolar lavage cell differential, we observed a significant strain-dependence in numbers of each of the lymphocyte, macrophage and neutrophil (PMN) cell types at 7 days post irradiation (p < 0.001), as shown in Figure 2. In addition, numbers of PMNs and macrophages also varied at the 6 h time point (p < 0.013) as shown in Figure 2. Control cell numbers and those at 1 day post radiation did not significantly differ among strains (p > 0.02). Radiation produced only minimal changes in bronchoalveolar cell number, when assayed up to 7 days post exposure, as illustrated in Additional file 5: Figure S5. One exception to this was the significantly altered cell numbers in lavage procured from AKR/J mice.

Analyses of these lavage cell data as predictive of lung disease, both singly and through multiple regression, revealed no significant correlations with either of the fibrosis score or the survival time (p > 0.09).

### Acute Bronchoalveolar lavage cytokine response to thoracic irradiation

**Figure 3:**
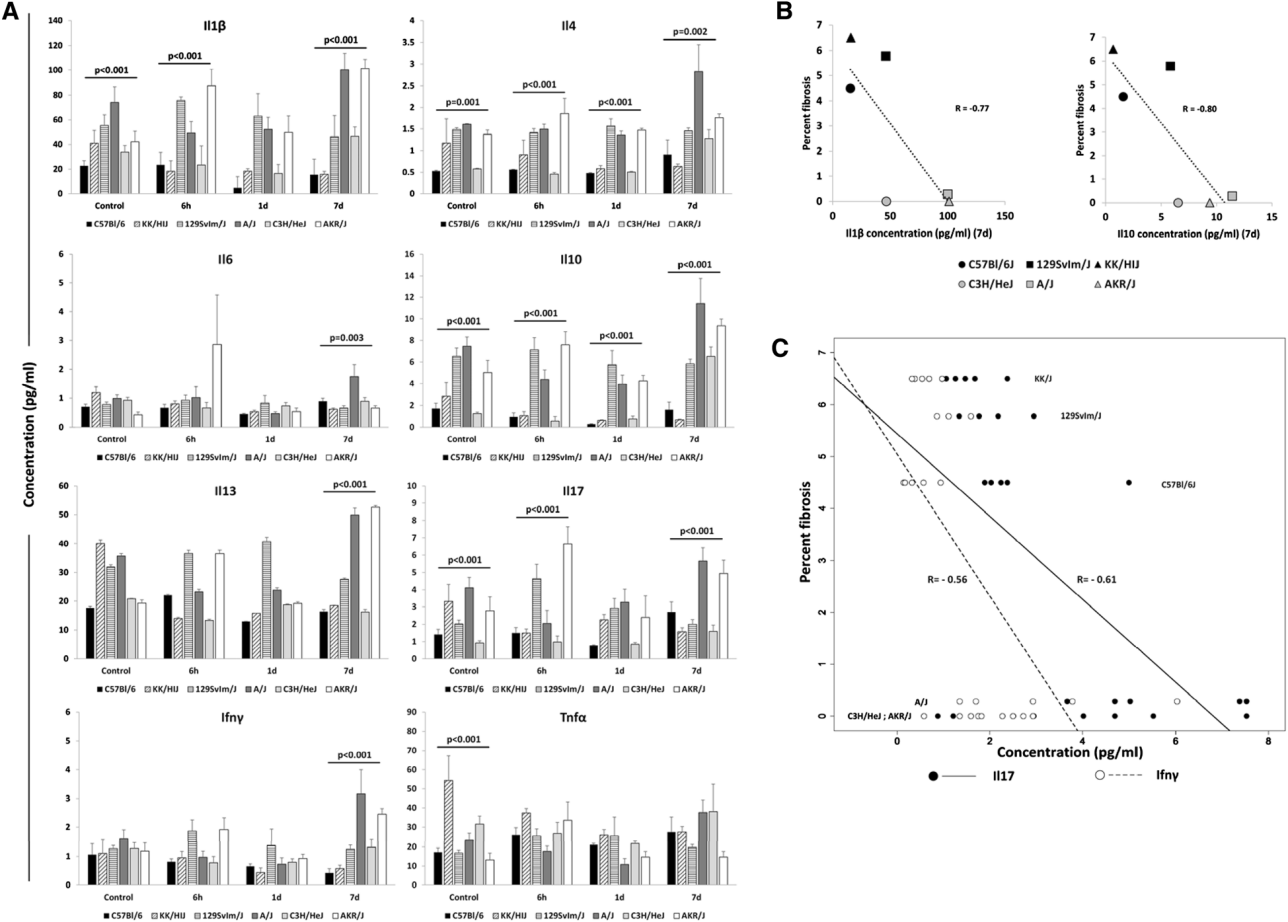
**Strain dependent bronchoalveolar lavage cytokine levels in acute radiation response.** Bronchoalveolar lavage fluid was obtained from control and irradiated mice at 6 hours, 1 day and 7 days post-irradiation. **A)** Bronchoalveolar lavage cytokine concentrations measured using the Multiplex assay. Results are presented as mean ± SE for groups of n = 4-5 mice. Significant variation in inbred strain measures by ANOVA indicated. **B)** Pearson correlation coefficients of late lung disease phenotypes with bronchoalveolar lavage cytokine levels (correlation coefficients between the fibrosis score as percent pulmonary fibrosis and the cytokine concentrations were R = −0.77, −0.80 and p-values 0.07, 0.05 respectively) **C)** Linear regression of the pulmonary fibrosis score against the lavage levels of Il17 and Ifnγ recorded at the day 7 time point. A significant interaction (p = 0.008) between the Il17 and Ifnγ measures is indicated by the intersecting regression lines of the fibrosis score against level of each cytokine.

To investigate whether irradiation induced a strain dependent pulmonary cytokine response, the levels of eight cytokines were profiled in bronchoalveolar lavage procured from each mouse at early time points post irradiation. Our selection includes cytokines previously associated with the lung response to radiation in both mice and humans such as Il6 and Tnfα [15,16,37,38]) as well as pulmonary fibrosis-promoting Il1β, Il13 and Il17 [39,40]) and anti-fibrotic mediators Il10 and Ifnγ [41]. Radiation exposure produced minimal changes in the cytokine profiles of these strains when assessed at acute time points (Additional file 6: Figure S6). Inbred strain background, however, significantly affected the cytokine level as shown in Figure 3A.

To evaluate whether cytokine levels predicted for the impending lung disease in the 6 inbred strains, we performed a correlation analysis with each of fibrosis score and survival time. As shown in Figure 3B Il1β and Il10, measured at the 7 day timepoint, were inversely correlated with fibrosis score (R = −0.77, p = 0.07; R = −0.80, p = 0.05), with marginal significance. Unlike the suggestive correlation to extent of pulmonary fibrosis, cytokine levels in the lavage were not significantly correlated with the trait of post irradiation survival time (p > 0.18, data not shown).

To investigate whether particular cytokine levels in the BAL, collectively, predicted for pulmonary fibrosis, we performed a multiple regression analysis. The combinations of Il17 and Ifnγ; Il17, Ifnγ and Il13; Tnfα, Il1β and Il6; Il6 and Il10; and Il6, Il10 and Il17 were tested. At the 7 day time point, the levels of Il17 and Ifnγ and their interaction explained a significant proportion of the variability (p = 0.001, 0.008 and 0.008 for Ifnγ, Il17 and the interaction term respectively), in fibrosis score, as shown in Figure 3C. The interaction model better explained the fibrosis score than the models of Il17 or Ifnγ levels alone: p = 0.005 and p = 0.01 respectively, by ANOVA). The addition of Il13 measures did not improve the model (p = 0.38). The lavage cytokine levels are inversely correlated with pulmonary fibrosis, as depicted in Figure 3C. No significant correlation was evident through multiple regression analysis between the remaining combinations of lavage cytokine levels and survival time (p > 0.1, data not shown).

### Acute Serum cytokine response to thoracic irradiation

**Figure 4:**
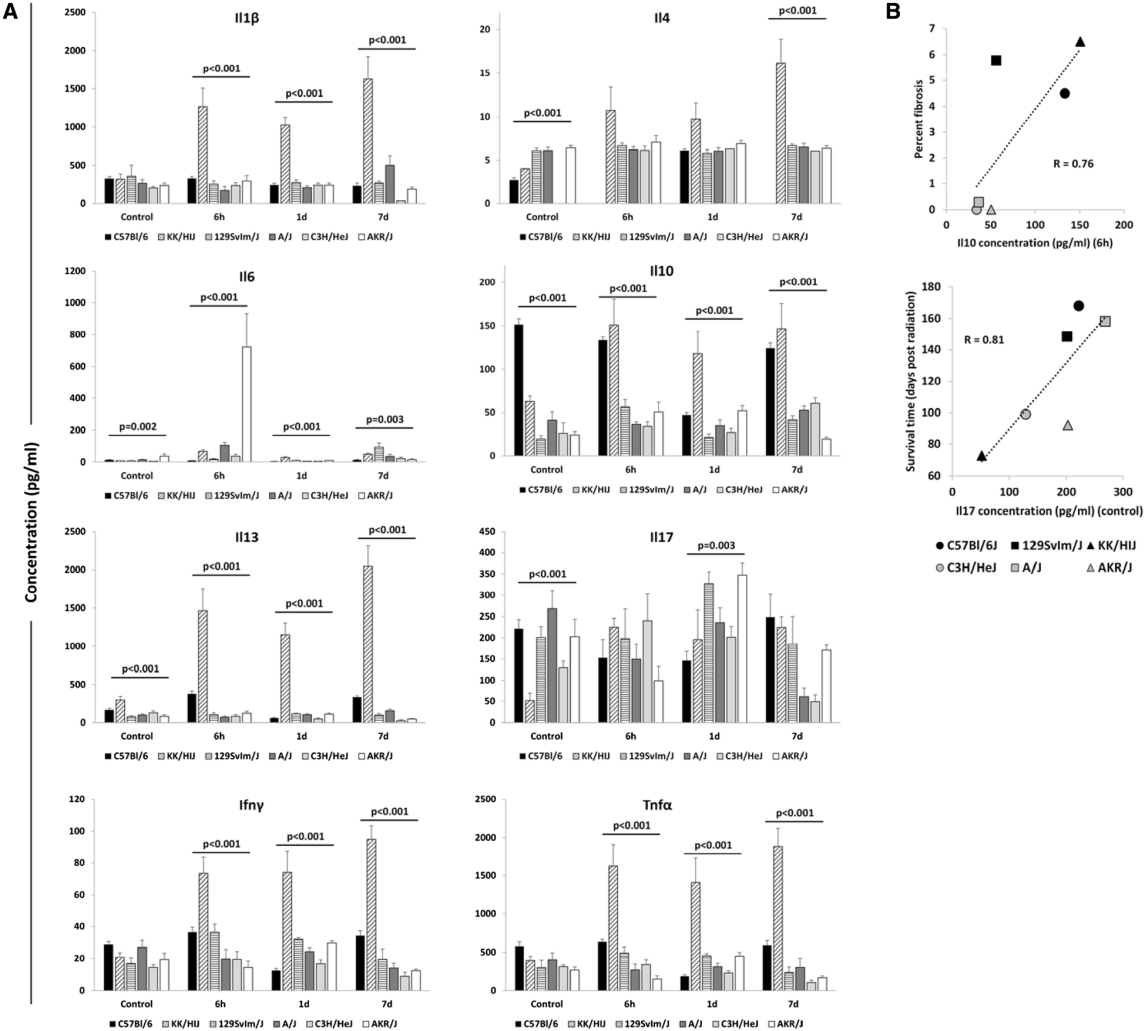
**Strain dependent serum cytokine levels in acute radiation response.** Serum was collected from mice at 6 hours, 1 day and 7 days post-irradiation. **A)** Cytokine concentrations measured using the Multiplex assay. Results are presented as mean ± SE for groups of n = 4-5 mice. Significant variation in inbred strain measures by ANOVA indicated. **B)** Pearson correlation of late disease phenotypes with serum cytokine levels (R = 0.76, p = 0.07 between percent fibrosis and Il10 concentration at 6 hours; R = 0.81, p = 0.04 between survival time and control Il17 concentration).

To determine whether the propensity to develop a lung disease phenotype was evident early on in a more clinically accessible tissue, serum cytokine levels were measured and are shown in Figure 4A. Serum cytokine levels of IL6, Il10 and Il13 were found to depend on strain at all assay times while a significant strain effect in Il1β, Ifnγ and Tnfα was observed for all time points with the exception of control. Radiation treatment also affected serum cytokine levels as presented in Additional file 7: Figure S7, with Il6, Il17 and Tnfα levels affected by radiation in 4 of six strains.

Presented in Figure 4B are the results of analyses to evaluate whether serum cytokine levels were correlated with later lung disease in these strains. As shown, the Il17 level in the serum of control mice was significantly correlated with survival time (R = 0.81, p = 0.04), and Il10 measures at 6 h (R = 0.76, p = 0.07) were suggestively correlated with fibrosis score.

By multiple linear regression analyses no significant correlations between combinations of serum cytokine levels, as described for lavage, and the phenotypes of fibrosis or survival time (p > 0.11, data not shown) were revealed.

## Discussion

When a combined regimen of radiotherapy and chemotherapy is used, the incidence of radiation pneumonitis can reach 40-60% [42,43], meaning that the implementation of more aggressive therapies for thoracic malignancies is hindered by a high risk of pulmonary side effects in the treated population. Moreover, patients prone to develop pulmonary fibrosis, an incurable condition arising following radiotherapy, at present cannot be identified prior to therapy [44]. Therefore the identification of cellular and molecular markers to predict a patient’s propensity to pulmonary adverse effects is paramount to more efficient cancer therapies.

Our approach builds on previous studies aimed at evaluating early tissue cytokine levels as markers predictive of late radiation side effects. Specifically, in this study we characterised the primary radiation injury response of the lung following thoracic irradiation in six inbred mouse strains in terms of infiltrating Thelper lymphocyte populations, bronchoalveolar lavage cell differentials, as well as serum and lavage cytokine measurements, and investigated whether any of these initial immune response markers predicted for the late disease phenotypes in the lung.

Our definition of the initial injury response as that occurring at 6 h, 1 day and 7 days post radiation administration is similar to that used in previous reports [45-47]. In addition, changes in the levels of specific cytokines after radiation agree with data from prior reports. For example, the decreased amounts of Il1β at 1 day post treatment in lavage from C3H/HeJ and C57Bl/6 mice and the lack of a radiation-induced change in amounts of Tnfα across this time period agree well with the findings of Hong et al. [46] who measured changes in cytokine mRNA in lung tissue from C3H/HeJ and C57Bl/6 mice. Secondly, Ao et al. [45] measured cytokine levels in the lung and serum of irradiated C3H/HeJ and C57Bl/6 mice, and Il6 was the only cytokine common to our study and theirs. Our findings for this cytokine agree with theirs for three of four time points, with the exception that we did not detect an increase in interleukin-6 at the 6 hours post irradiation in C57Bl/6 mice as they reported. Finally, our data are supported by a report characterising the lymphocyte response of C57Bl/6 mice to whole thorax irradiation wherein radiation-induced changes in cell numbers were evident in the lungs of mice at 6 weeks following therapy, but not at 10 days, which is similar to our 7d data set in these mice [26].

Of the potential immune and inflammatory biomarkers profiled, our analyses revealed serum Il17 levels in untreated mice and Th1 cell numbers at 7 days post exposure to best correlate with the onset of radiation induced respiratory distress. Given that each of the six strains evaluated here succumbs to pneumonitis, with or without fibrosis, after high dose whole thorax irradiation we chose survival time differences to reflect pneumonitis. These putative associations, therefore, if experimentally confirmed, suggest that differing adaptive immune biases may exist in mice and affect an early versus late development of pneumonitis after radiation exposure. Supporting this are findings from mice, where a thymectomy was found to decrease the incidence of radiation pneumonitis [48,49], and radiation-induced changes in regulatory T cell numbers to potentially influence the lung inflammatory response [26].

In the six inbred strains evaluated here, the extent of pulmonary fibrosis was associated with a specific adaptive immune response both by tissue lymphocyte counts and bronchoalveolar lavage cytokine levels. Specifically, at 7 days post irradiation, a linear combination of Th1 and Th17 cell numbers was revealed to be a significant predictor for the degree of pulmonary fibrosis and, at the same time point, we observed the combination of Il17 and Ifnγ levels in the bronchoalveolar lavage to significantly associate with late stage fibrosis. That these associations were evident at the 7 day timepoint and not earlier, is consistent with the delay required for the activation of the adaptive immune response. Further, at the 7 day time point, we measured the highest numbers of infiltrating Th17 cells in the lungs of KK/HIJ mice and this response supported the correlation of lymphocytes with fibrosis identified. Among the fibrosis-prone strains, KK/HIJ mice are the first to succumb to lung disease (at 8 weeks post irradiation, compared to C57BL/6J and 129/SvImJ mice which succumb at 23 and 26 weeks respectively) and thus the Th17 subset cell count evident at day 7 may be both correlated to fibrosis and related to an accelerated development of fibrosis in this strain.

Il17 and Ifnγ have been reported to exert opposing effects on the development of pulmonary fibrosis [50-53] and a predictive model based on their interaction supports the paradigm that fibrotic lung disease is the result of the synergy between various immune cell types and their cytokine repertoires [54]. The finding of fibrosis score as negatively correlated with the Il17 and Ifnγ cytokine levels, but positively correlated with Th17 and Th1 cells may be related to the observation that bronchoalveolar lavage and interstitial cells have different roles in radiation-induced lung injury [55], or may indicate the occurrence of a negative feedback loop influencing these pulmonary responses.

Further to interferonγ and interleukin-17, there was more interleukin 1β and interleukin 10 in the lavage of mice which would later succumb to pneumonitis alone compared to those succumbing to pneumonitis with fibrosis. That higher levels interleukin 1β would be associated with decreased fibrosis, appears in contradiction with the literature which demonstrates this cytokine to promote pulmonary fibrosis [53] and to mediate radiation-induced skin fibrosis [56]. Similarly, interleukin 10 is considered anti-inflammatory [57] thus higher levels of this cytokine in strains developing the inflammatory response of pneumonitis would be unexpected. These correlations may thus suggest that the same cytokine could have opposing roles in the early versus the late stages of radiation-induced lung disease. Along this line, the wave-like pattern of the molecular responses in irradiated tissues [37,58] raises the question of whether the early phase of cytokines is merely the indicator of the inflammatory process or is causally linked with the late stages of disease.

The severity of pulmonary fibrosis was also associated with levels of a particular cytokine in the serum and, as with the bronchoalveolar lavage, the correlation may be to the disease but not necessarily to the disease process. In detail, increases in serum interleukin 10, measured at the 6 hour time point, were correlated with greater fibrosis score in mice. As we also determined interleukin 10 in the lavage to be associated with reduced fibrosis the serum and lavage do not appear to be reflecting the same process. This finding is in contrast to those of Ao et al. [45], who reported a correlation between serum and lavage cytokine concentrations in lungs of irradiated C57BL/6J and C3H/HeJ mice. This correlation was not evident in the panel of six strains evaluated in the present work.

Our approach is strengthened by the use of six inbred strains with diverse phenotypes in terms of lung disease [22], and offers a more robust evaluation of biomarkers, compared to studies using two inbred strains [30,37,45-47], although it also includes specific limitations with regard to potential clinical extrapolation. The lung phenotypes studied here are in response to 18 Gy irradiation, a dose which was chosen to elicit respiratory distress in the vast majority of mice within 6 months, for the purpose of characterizing the pneumonitis or pneumonitis with fibrosis response of each strain. This dose, although useful in producing clinically relevant lung phenotypes in mice, is not often used clinically, apart from stereotactic radiotherapy, thus the correlations revealed here are valid within the model system and useful for directing clinical investigation. Secondly, the anaesthesia given to the irradiated mice may have been immunomodulatory and thus could affect the phenotypes reported here. The strengths of the model system include the ability to control the environment, which is especially relevant for lung disease responses, to eliminate confounding patient and cancer treatment related factors, and to permit evaluation of biological replicates. The identification of predictive markers for murine lung disease therefore represents a starting point for subsequent human studies.

## Conclusions

By evaluating the responses of 6 inbred mouse strains which provided genetic background diversity in a controlled environmental setting, we identified correlations of lower Il17 and Ifnγ values in lavage as well as increased numbers of infiltrating Th1 and Th17 cells with fibrosis severity. Additional associations were found between particular lavage and serum cytokines with fibrosis severity and survival time, but no evidence was found to support that numbers of lavage macrophages, lymphocytes or neutrophils are relevant early response markers in determining the course of late-stage lung disease.

## Additional files

Additional file 1: Figure S1. Gating strategy to identify pulmonary T helper cell populations. Mice were exposed to 18 Gy of thorax irradiation and euthanized at 6 hours, 1 day and 7 days post treatment. Lung tissue was dispersed and stained with antibodies against CD4, Ifnγ, Il13 and Il17. Lymphocytes were gated on the FSC/SSC plot and numbers of Th1/2/17 cells were determined among the CD4+ lymphocytes. Additional file 2: Figure S2. Effect of whole thorax irradiation on pulmonary T helper cell populations. Data of Figure 1 grouped by the fibrosis susceptible (KK/HIJ, C57BL/6J, 129S1/SvImJ) and fibrosis resistant (C3H/HeJ, A/J and AKR/J) strains. Significant variation in measures over time indicated by ANOVA. Additional file 3: Figure S3. Correlation of late lung disease phenotypes with acute response pulmonary T helper cell populations. Mice were exposed to 18 Gy of thorax irradiation, euthanized at 6 hours, 1 day and 7 days post treatment and T helper cells were ennumerated through flow cytometry of total lung tissue. Pearson correlation tests were performed between the lung T helper cell populations and late stage fibrosis score and post irradiation survival time (R = 0.75, p = 0.08 for survival time with CD4 + Ifnγ + cells at 7 days, and R = −0.71, p = 0.1 for late stage fibrosis score and CD4 + Il13+ cells recorded at 6 hours). Additional file 4: Figure S4. Post irradiation bronchoalveolar lavage cell counts and correlation to late stage fibrosis. Following exposure to 18 Gy of thorax irradiation, bronchoalveolar lavage was collected from irradiated mice at 6 hours, 1 day and 7 days time points. A) Total cell counts per millilitre of lavage fluid. Results are presented as mean ± SE for groups of n = 4–5 mice. Significant variation in inbred strain measures by ANOVA indicated; B) Pearson correlation of total cell numbers in lavage with fibrosis score in the 6 inbred strains of mice (R = 0.77, p = 0.07). Additional file 5: Figure S5. Effect of whole thorax irradiation on bronchoalveolar lavage cell differentials. Data of Figure 2 grouped by the fibrosis susceptible (KK/HIJ, C57BL/6J, 129S1/SvImJ) and fibrosis resistant (C3H/HeJ, A/J and AKR/J) strains. Significant variation in measures over time indicated by ANOVA. Additional file 6: Figure S6. Effect of whole thorax irradiation on cytokine levels in bronchoalveolar lavage. Data of Figure 3 grouped by the fibrosis susceptible (KK/HIJ, C57BL/6J, 129S1/SvImJ) and fibrosis resistant (C3H/HeJ, A/J and AKR/J) strains. Significant variation in measures over time indicated by ANOVA. Additional file 7: Figure S7. Effect of whole thorax irradiation on serum cytokine levels. Data of Figure 4 grouped by the fibrosis susceptible (KK/HIJ, C57BL/6J, 129S1/SvImJ) and fibrosis resistant (C3H/HeJ, A/J and AKR/J) strains. Significant variation in measures over time indicated by ANOVA.

## References

[1] Osterreicher J, Pejchal J, Skopek J, Mokry J, Vilasova Z, Psutka J (2004). Role of type II pneumocytes in pathogenesis of radiation pneumonitis: dose response of radiation-induced lung changes in the transient high vascular permeability period. Exp Toxicol Pathol.

[2] Van der Meeren A, Vandamme M, Squiban C, Gaugler MH, Mouthon MA (2003). Inflammatory reaction and changes in expression of coagulation proteins on lung endothelial cells after total-body irradiation in mice. Radiat Res.

[3] Roberts CM, Foulcher E, Zaunders JJ, Bryant DH, Freund J, Cairns D (1993). Radiation pneumonitis: a possible lymphocyte-mediated hypersensitivity reaction. Ann Intern Med.

[4] Takigawa N, Segawa Y, Saeki T, Kataoka M, Ida M, Kishino D (2000). Bronchiolitis obliterans organizing pneumonia syndrome in breast-conserving therapy for early breast cancer: radiation-induced lung toxicity. Int J Radiat Oncol Biol Phys.

[5] Rube CE, Uthe D, Wilfert F, Ludwig D, Yang K, Konig J (2005). The bronchiolar epithelium as a prominent source of pro-inflammatory cytokines after lung irradiation. Int J Radiat Oncol Biol Phys.

[6] Kuwano K, Hagimoto N, Nakanishi Y (2004). The role of apoptosis in pulmonary fibrosis. Histol Histopathol.

[7] Abe S, Boyer C, Liu X, Wen FQ, Kobayashi T, Fang Q (2004). Cells derived from the circulation contribute to the repair of lung injury. Am J Respir Crit Care Med.

[8] Epperly MW, Guo H, Gretton JE, Greenberger JS (2003). Bone marrow origin of myofibroblasts in irradiation pulmonary fibrosis. Am J Respir Cell Mol Biol.

[9] Russell NS, Grummels A, Hart AA, Smolders IJ, Borger J, Bartelink H (1998). Low predictive value of intrinsic fibroblast radiosensitivity for fibrosis development following radiotherapy for breast cancer. Int J Radiat Biol.

[10] Peacock J, Ashton A, Bliss J, Bush C, Eady J, Jackson C (2000). Cellular radiosensitivity and complication risk after curative radiotherapy. Radiother Oncol.

[11] Brock WA, Tucker SL, Geara FB, Turesson I, Wike J, Nyman J (1995). Fibroblast radiosensitivity versus acute and late normal skin responses in patients treated for breast cancer. Int J Radiat Oncol Biol Phys.

[12] Burnet NG, Nyman J, Turesson I, Wurm R, Yarnold JR, Peacock JH (1992). Prediction of normal-tissue tolerance to radiotherapy from in-vitro cellular radiation sensitivity. Lancet.

[13] Herskind C, Bamberg M, Rodemann HP (1998). The role of cytokines in the development of normal-tissue reactions after radiotherapy. Strahlenther Onkol.

[14] Chen Y, Williams J, Ding I, Hernady E, Liu W, Smudzin T (2002). Radiation pneumonitis and early circulatory cytokine markers. Semin Radiat Oncol.

[15] Chen Y, Hyrien O, Williams J, Okunieff P, Smudzin T, Rubin P (2005). Interleukin (IL)-1A and IL-6: applications to the predictive diagnostic testing of radiation pneumonitis. Int J Radiat Oncol Biol Phys.

[16] Arpin D, Perol D, Blay JY, Falchero L, Claude L, Vuillermoz-Blas S (2005). Early variations of circulating interleukin-6 and interleukin-10 levels during thoracic radiotherapy are predictive for radiation pneumonitis. J Clin Oncol.

[17] Stenmark MH, Cai XW, Shedden K, Hayman JA, Yuan S, Ritter T (2012). Combining physical and biologic parameters to predict radiation-induced lung toxicity in patients with non-small-cell lung cancer treated with definitive radiation therapy. Int J Radiat Oncol Biol Phys.

[18] Rubin P, Johnston CJ, Williams JP, McDonald S, Finkelstein JN (1995). A perpetual cascade of cytokines postirradiation leads to pulmonary fibrosis. Int J Radiat Oncol Biol Phys.

[19] McBride WH (1995). Cytokine cascades in late normal tissue radiation responses. Int J Radiat Oncol Biol Phys.

[20] Sharplin J, Franko AJ (1989). A quantitative histological study of strain-dependent differences in the effects of irradiation on mouse lung during the intermediate and late phases. Radiat Res.

[21] Moore BB, Hogaboam CM (2008). Murine models of pulmonary fibrosis. Am J Physiol Lung Cell Mol Physiol.

[22] Paun A, Haston CK (2012). Genomic and genome-wide association of susceptibility to radiation-induced fibrotic lung disease in mice. Radiother Oncol.

[23] Thomas DM, Fox J, Haston CK (2010). Imatinib therapy reduces radiation-induced pulmonary mast cell influx and delays lung disease in the mouse. Int J Radiat Biol.

[24] Johnston CJ, Williams JP, Elder A, Hernady E, Finkelstein JN (2004). Inflammatory cell recruitment following thoracic irradiation. Exp Lung Res.

[25] Haston CK, Begin M, Dorion G, Cory SM (2007). Distinct loci influence radiation-induced alveolitis from fibrosing alveolitis in the mouse. Cancer Res.

[26] Wirsdörfer F, Cappuccini F, Niazman M, de Leve S, Westendorf AM, Lüdemann L (2014). Thorax irradiation triggers a local and systemic accumulation of immunosuppressive CD4+ FoxP3+ regulatory T cells. Radiat Oncol.

[27] Toma CL, Serbescu A, Alexe M, Cervis L, Ionita D, Bogdan MA (2010). The bronchoalveolar lavage pattern in radiation pneumonitis secondary to radiotherapy for breast cancer. Maedica (Buchar).

[28] Han G, Zhang H, Xie CH, Zhou YF (2011). Th2-like immune response in radiation-induced lung fibrosis. Oncol Rep.

[29] Weaver CT, Elson CO, Fouser LA, Kolls JK (2013). The Th17 pathway and inflammatory diseases of the intestines, lungs, and skin. Annu Rev Pathol.

[30] Chiang CS, Liu WC, Jung SM, Chen FH, Wu CR, McBride WH (2005). Compartmental responses after thoracic irradiation of mice: strain differences. Int J Radiat Oncol Biol Phys.

[31] Paun A, Lemay AM, Haston CK (2010). Gene expression profiling distinguishes radiation-induced fibrosing alveolitis from alveolitis in mice. Radiat Res.

[32] Hackstein H, Wachtendorf A, Kranz S, Lohmeyer J, Bein G, Baal N (2012). Heterogeneity of respiratory dendritic cell subsets and lymphocyte populations in inbred mouse strains. Respir Res.

[33] O'Brien TJ, Letuve S, Haston CK (2005). Radiation-induced strain differences in mouse alveolar inflammatory cell apoptosis. Can J Physiol Pharmacol.

[34] Lemay AM, Haston CK (2008). Radiation-induced lung response of AcB/BcA recombinant congenic mice. Radiat Res.

[35] Fox J, Gordon JR, Haston CK (2011). Combined CXCR1/CXCR2 antagonism decreases radiation-induced alveolitis in the mouse. Radiat Res.

[36] Paun A, Fox J, Balloy V, Chignard M, Qureshi ST, Haston CK (2010). Combined Tlr2 and Tlr4 deficiency increases radiation-induced pulmonary fibrosis in mice. Int J Radiat Oncol Biol Phys.

[37] Rube CE, Wilfert F, Palm J, Konig J, Burdak-Rothkamm S, Liu L (2004). Irradiation induces a biphasic expression of pro-inflammatory cytokines in the lung. Strahlenther Onkol.

[38] Barthelemy-Brichant N, Bosquee L, Cataldo D, Corhay JL, Gustin M, Seidel L (2004). Increased IL-6 and TGF-beta1 concentrations in bronchoalveolar lavage fluid associated with thoracic radiotherapy. Int J Radiat Oncol Biol Phys.

[39] Gasse P, Riteau N, Vacher R, Michel ML, Fautrel A, di Padova F (2011). IL-1 and IL-23 mediate early IL-17A production in pulmonary inflammation leading to late fibrosis. PLoS One.

[40] Westermann W, Schobl R, Rieber EP, Frank KH (1999). Th2 cells as effectors in postirradiation pulmonary damage preceding fibrosis in the rat. Int J Radiat Biol.

[41] Nakagome K, Dohi M, Okunishi K, Tanaka R, Miyazaki J, Yamamoto K (2006). In vivo IL-10 gene delivery attenuates bleomycin induced pulmonary fibrosis by inhibiting the production and activation of TGF-beta in the lung. Thorax.

[42] Antonadou D, Coliarakis N, Synodinou M, Athanassiou H, Kouveli A, Verigos C (2001). Randomized phase III trial of radiation treatment +/− amifostine in patients with advanced-stage lung cancer. Int J Radiat Oncol Biol Phys.

[43] Reckzeh B, Merte H, Pfluger KH, Pfab R, Wolf M, Havemann K (1996). Severe lymphocytopenia and interstitial pneumonia in patients treated with paclitaxel and simultaneous radiotherapy for non-small-cell lung cancer. J Clin Oncol.

[44] Graves PR, Siddiqui F, Anscher MS, Movsas B (2010). Radiation pulmonary toxicity: from mechanisms to management. Semin Radiat Oncol.

[45] Ao X, Zhao L, Davis MA, Lubman DM, Lawrence TS, Kong FM (2009). Radiation produces differential changes in cytokine profiles in radiation lung fibrosis sensitive and resistant mice. J Hematol Oncol.

[46] Hong JH, Chiang CS, Tsao CY, Lin PY, McBride WH, Wu CJ (1999). Rapid induction of cytokine gene expression in the lung after single and fractionated doses of radiation. Int J Radiat Biol.

[47] Johnston CJ, Hernady E, Reed C, Thurston SW, Finkelstein JN, Williams JP (2010). Early alterations in cytokine expression in adult compared to developing lung in mice after radiation exposure. Radiat Res.

[48] McBride WH, Vegesna V (2000). The role of T-cells in radiation pneumonitis after bone marrow transplantation. Int J Radiat Biol.

[49] McBride WH, Vegesna V (1997). Role of the thymus in radiation-induced lung damage after bone marrow transplantation. Radiat Res.

[50] Gurujeyalakshmi G, Giri SN (1995). Molecular mechanisms of antifibrotic effect of interferon gamma in bleomycin-mouse model of lung fibrosis: downregulation of TGF-beta and procollagen I and III gene expression. Exp Lung Res.

[51] Kimura T, Ishii Y, Morishima Y, Shibuya A, Shibuya K, Taniguchi M (2004). Treatment with alpha-galactosylceramide attenuates the development of bleomycin-induced pulmonary fibrosis. J Immunol.

[52] Pochetuhen K, Luzina IG, Lockatell V, Choi J, Todd NW, Atamas SP (2007). Complex regulation of pulmonary inflammation and fibrosis by CCL18. Am J Pathol.

[53] Wilson MS, Madala SK, Ramalingam TR, Gochuico BR, Rosas IO, Cheever AW (2010). Bleomycin and IL-1beta-mediated pulmonary fibrosis is IL-17A dependent. J Exp Med.

[54] Lo Re S, Lison D, Huaux F (2013). CD4+ T lymphocytes in lung fibrosis: diverse subsets, diverse functions. J Leukoc Biol.

[55] Hong JH, Jung SM, Tsao TC, Wu CJ, Lee CY, Chen FH (2003). Bronchoalveolar lavage and interstitial cells have different roles in radiation-induced lung injury. Int J Radiat Biol.

[56] Liu W, Ding I, Chen K, Olschowka J, Xu J, Hu D (2006). Interleukin 1beta (IL1B) signaling is a critical component of radiation-induced skin fibrosis. Radiat Res.

[57] Sabat R, Grutz G, Warszawska K, Kirsch S, Witte E, Wolk K (2010). Biology of interleukin-10. Cytokine Growth Factor Rev.

[58] McBride WH, Chiang CS, Olson JL, Wang CC, Hong JH, Pajonk F (2004). A sense of danger from radiation. Radiat Res.

