# Supplementary figures and images for: Acute adaptive immune response correlates with late radiation-induced pulmonary fibrosis in mice
Source: Radiat Oncol. 2015 Feb 20;10:45. doi: 10.1186/s13014-015-0359-y (PMC4342202; doi:10.1186/s13014-015-0359-y)

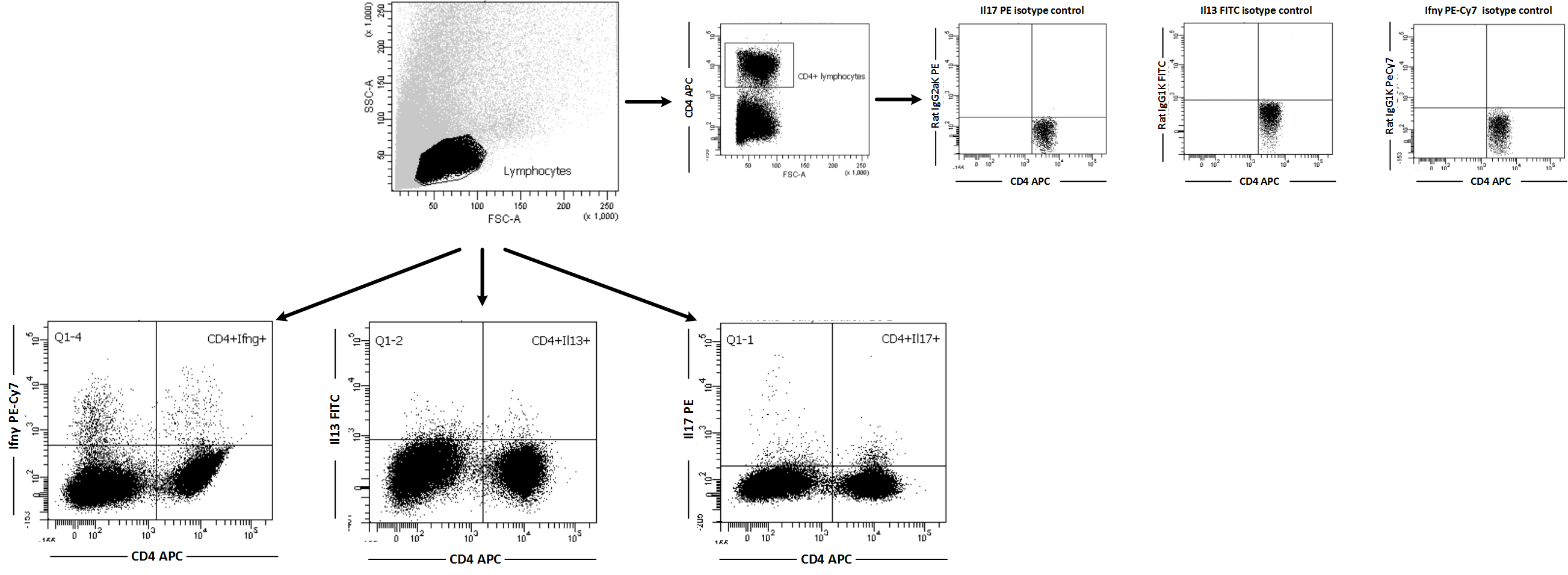

Supplement: Additional file 1: Figure S1. — Gating strategy to identify pulmonary T helper cell populations. Mice were exposed to 18 Gy of thorax irradiation and euthanized at 6 hours, 1 day and 7 days post treatment. Lung tissue was dispersed and stained with antibodies against CD4, Ifnγ, Il13 and Il17. Lymphocytes were gated on the FSC/SSC plot and numbers of Th1/2/17 cells were determined among the CD4+ lymphocytes. [file 13014_2015_359_MOESM1_ESM.png]

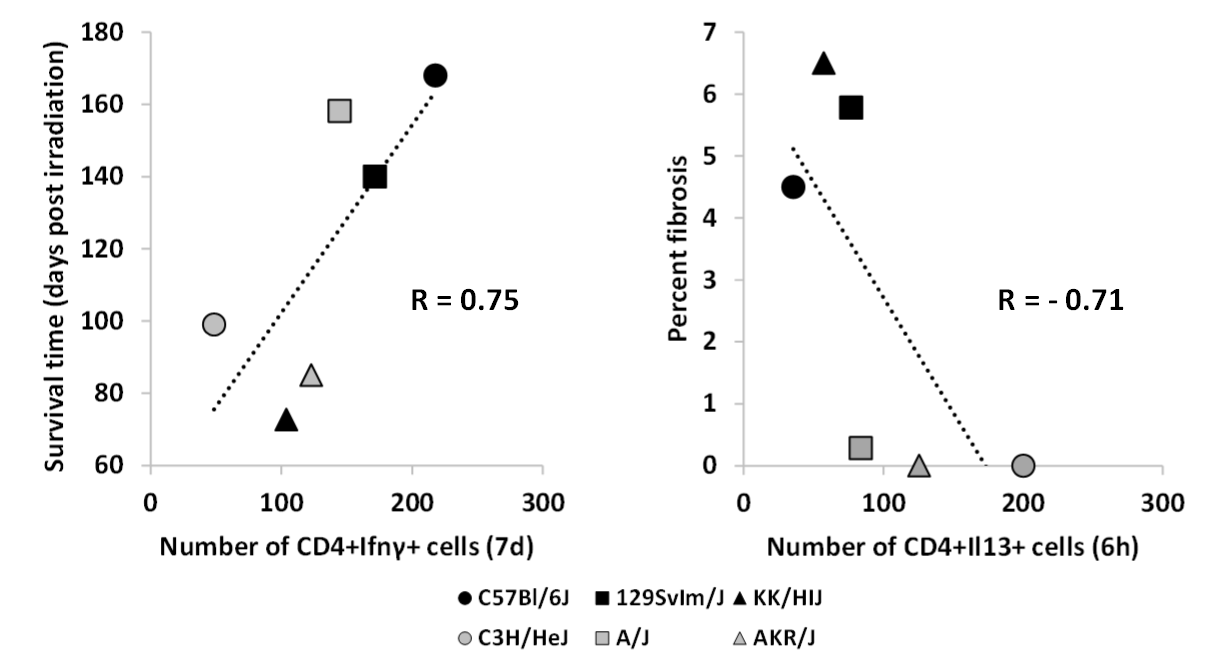

Supplement: Additional file 3: Figure S3. — Correlation of late lung disease phenotypes with acute response pulmonary T helper cell populations. Mice were exposed to 18 Gy of thorax irradiation, euthanized at 6 hours, 1 day and 7 days post treatment and T helper cells were ennumerated through flow cytometry of total lung tissue. Pearson correlation tests were performed between the lung T helper cell populations and late stage fibrosis score and post irradiation survival time (R = 0.75, p = 0.08 for survival time with CD4 + Ifnγ + cells at 7 days, and R = −0.71, p = 0.1 for late stage fibrosis score and CD4 + Il13+ cells recorded at 6 hours). [file 13014_2015_359_MOESM3_ESM.png]

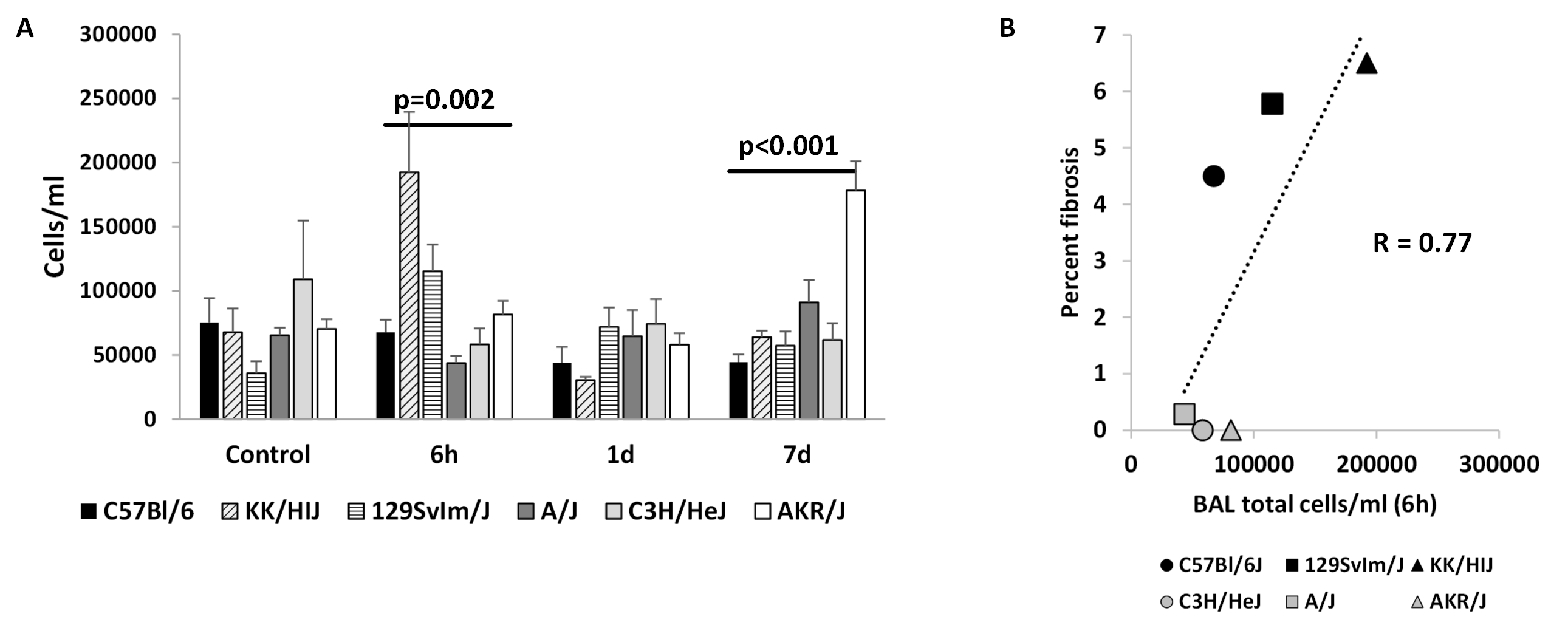

Supplement: Additional file 4: Figure S4. — Post irradiation bronchoalveolar lavage cell counts and correlation to late stage fibrosis. Following exposure to 18 Gy of thorax irradiation, bronchoalveolar lavage was collected from irradiated mice at 6 hours, 1 day and 7 days time points. A) Total cell counts per millilitre of lavage fluid. Results are presented as mean ± SE for groups of n = 4–5 mice. Significant variation in inbred strain measures by ANOVA indicated; B) Pearson correlation of total cell numbers in lavage with fibrosis score in the 6 inbred strains of mice (R = 0.77, p = 0.07). [file 13014_2015_359_MOESM4_ESM.png]

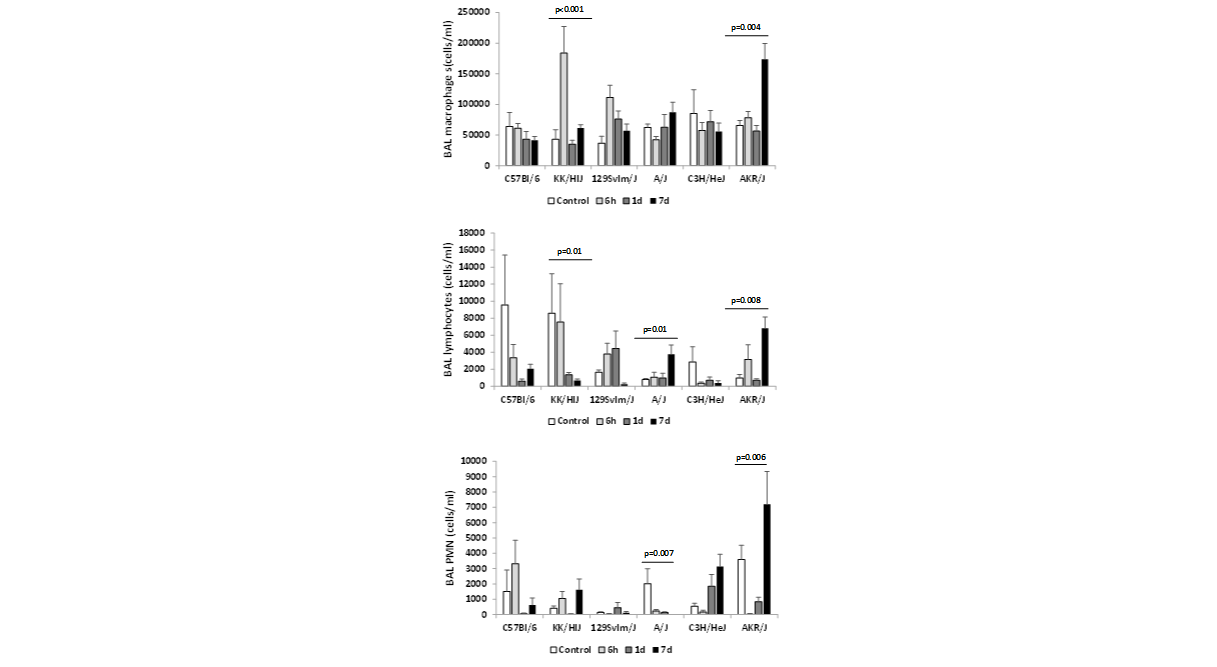

Supplement: Additional file 5: Figure S5. — Effect of whole thorax irradiation on bronchoalveolar lavage cell differentials. Data of Figure 2 grouped by the fibrosis susceptible (KK/HIJ, C57BL/6J, 129S1/SvImJ) and fibrosis resistant (C3H/HeJ, A/J and AKR/J) strains. Significant variation in measures over time indicated by ANOVA. [file 13014_2015_359_MOESM5_ESM.png]

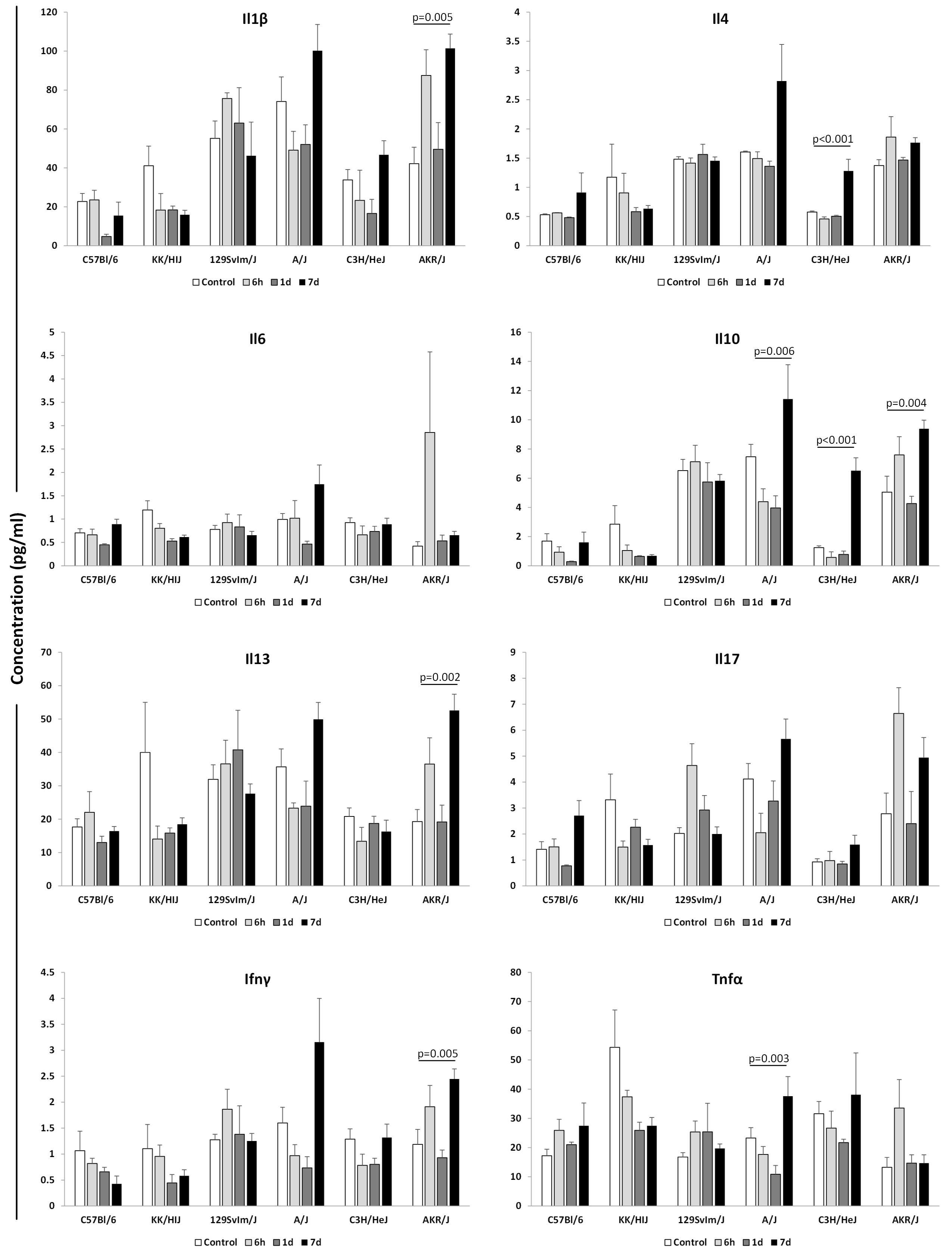

Supplement: Additional file 6: Figure S6. — Effect of whole thorax irradiation on cytokine levels in bronchoalveolar lavage. Data of Figure 3 grouped by the fibrosis susceptible (KK/HIJ, C57BL/6J, 129S1/SvImJ) and fibrosis resistant (C3H/HeJ, A/J and AKR/J) strains. Significant variation in measures over time indicated by ANOVA. [file 13014_2015_359_MOESM6_ESM.jpeg]

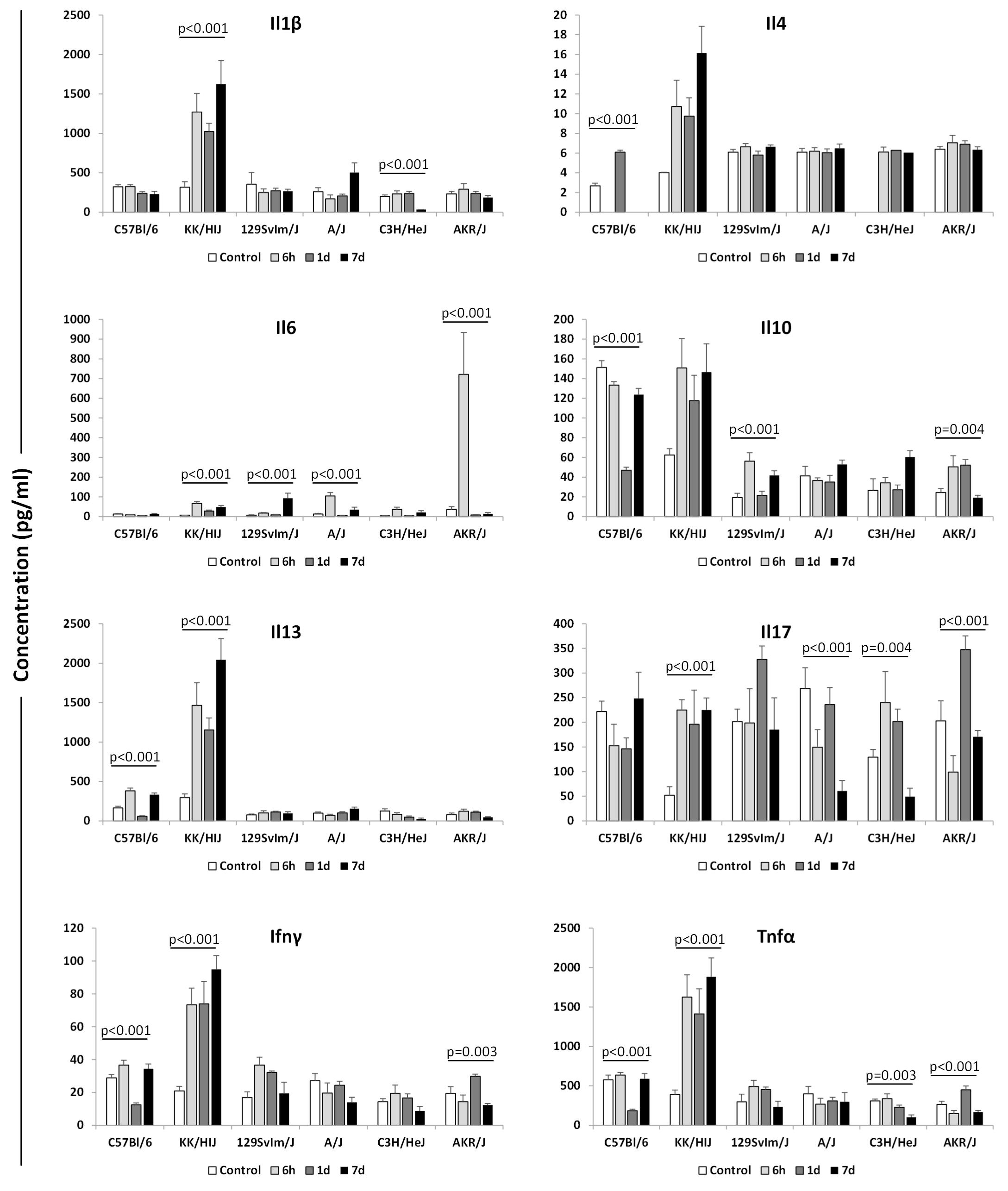

Supplement: Additional file 7: Figure S7. — Effect of whole thorax irradiation on serum cytokine levels. Data of Figure 4 grouped by the fibrosis susceptible (KK/HIJ, C57BL/6J, 129S1/SvImJ) and fibrosis resistant (C3H/HeJ, A/J and AKR/J) strains. Significant variation in measures over time indicated by ANOVA. [file 13014_2015_359_MOESM7_ESM.jpeg]
